# Sulfated Metabolites of Polychlorinated Biphenyls Are High-Affinity Ligands for the Thyroid Hormone Transport Protein Transthyretin

**DOI:** 10.1289/ehp.1206198

**Published:** 2013-04-12

**Authors:** Fabian A. Grimm, Hans-Joachim Lehmler, Xianran He, Larry W. Robertson, Michael W. Duffel

**Affiliations:** 1 Interdisciplinary Graduate Program in Human Toxicology,; 2 Department of Pharmaceutical Sciences and Experimental Therapeutics, College of Pharmacy, and; 3 Department of Occupational and Environmental Health, College of Public Health, The University of Iowa, Iowa City, Iowa, USA

**Keywords:** hydroxylated PCB, OH-PCB, PCB, PCB sulfates, polychlorinated biphenyl, sulfation, thyroid disruption, transthyretin

## Abstract

Background: The displacement of l-thyroxine (T_4_) from binding sites on transthyretin (TTR) is considered a significant contributing mechanism in polychlorinated biphenyl (PCB)-induced thyroid disruption. Previous research has discovered hydroxylated PCB metabolites (OH-PCBs) as high-affinity ligands for TTR, but the binding potential of conjugated PCB metabolites such as PCB sulfates has not been explored.

Objectives: We evaluated the binding of five lower-chlorinated PCB sulfates to human TTR and compared their binding characteristics to those determined for their OH-PCB precursors and for T_4_.

Methods: We used fluorescence probe displacement studies and molecular docking simulations to characterize the binding of PCB sulfates to TTR. The stability of PCB sulfates and the reversibility of these interactions were characterized by HPLC analysis of PCB sulfates after their binding to TTR. The ability of OH-PCBs to serve as substrates for human cytosolic sulfotransferase 1A1 (hSULT1A1) was assessed by OH-PCB–dependent formation of adenosine-3´,5´-diphosphate, an end product of the sulfation reaction.

Results: All five PCB sulfates were able to bind to the high-affinity binding site of TTR with equilibrium dissociation constants (*K*_d_ values) in the low nanomolar range (4.8–16.8 nM), similar to that observed for T_4_ (4.7 nM). Docking simulations provided corroborating evidence for these binding interactions and indicated multiple high-affinity modes of binding. All OH-PCB precursors for these sulfates were found to be substrates for hSULT1A1.

Conclusions: Our findings show that PCB sulfates are high-affinity ligands for human TTR and therefore indicate, for the first time, a potential relevance for these metabolites in PCB-induced thyroid disruption.

Polychlorinated biphenyls (PCBs) comprise a group of 209 former industrial chemicals that are classified as toxic, bioaccumulative, and environmentally persistent (Safe 1993). Many PCBs, particularly the lower-chlorinated PCB congeners (LC-PCBs), possess semivolatile properties and are detected globally in air, with the highest concentrations being found in industrialized urban areas (Breivik et al. 2007; Hu et al. 2010; Wethington and Hornbuckle 2005). In contrast to the higher-chlorinated congeners, LC-PCBs are more susceptible to metabolic conversion, but their fate and toxicities within the human body remain relatively poorly understood (Robertson and Ludewig 2011). Considering the abundance of LC-PCBs, and the resulting public health concerns, the identification of their toxicologically relevant metabolites and their molecular targets is essential for risk assessment purposes. To date, research has been primarily focused on the toxicological effects of parent PCBs and their hydroxylated metabolites (OH-PCBs).

Certain OH-PCBs have been identified as excellent substrates for cytosolic sulfotransferases (SULTs), a family of enzymes that catalyze the formation of sulfate conjugates (Ekuase et al. 2011; Liu et al. 2009; van den Hurk et al. 2002; Wang et al. 2006). Dhakal et al. (2012) recently reported that the formation of sulfate conjugates is a major metabolic pathway for PCB 3 in rats, thereby providing evidence for the formation of PCB sulfates *in vivo*. Despite these indications of the potential for the formation of PCB sulfates *in vivo*, their fates and toxicities have not yet been characterized.

The thyroid gland is a target of PCB toxicity. Chronic exposure to commercial PCB mixtures increases the mass of the thyroid gland and the number of thyroid neoplasms (Mayes et al. 1998). Both of these changes may be linked to a PCB-driven reduction in serum levels of thyroid hormones (Knerr and Schrenk 2006), a commonly measured result of PCB exposure (Pearce and Braverman 2009). Parent PCBs and OH-PCBs are known thyroid disruptors that induce multiple effects on the thyroid homeostatic system in a congener-dependent manner (Boas et al. 2009; Brouwer et al. 1999; Patrick 2009). Epidemiological studies have revealed a correlation between prenatal PCB exposure and behavioral effects, decreased cognitive function, and mental retardation in infants, and these effects may be caused by PCB-induced alterations of the thyroid status in the fetal brain (Boucher et al. 2009; Darras 2008; Schantz and Widholm 2001). A significant contributing mechanism in PCB-induced hypothyroxinemia is the displacement of l-thyroxine (T_4_) from its binding sites on the thyroid hormone transport protein transthyretin (TTR) (Gutleb et al. 2010; Kodavanti and Curras-Collazo 2010; Ucan-Marin et al. 2009, 2010). TTR is the main transporter of T_4_ in the cerebrospinal fluid and is, in addition to serum albumin and thyroxine-binding globulin, one of three thyroid hormone carriers in human plasma (Larsson et al. 1985; Petitpas et al. 2003). Certain OH-PCBs were previously identified as ligands for TTR that were capable of competing with T_4_ for its two binding sites (Lans et al. 1993; Purkey et al. 2004; Rickenbacher et al. 1986). These sites exhibit negative cooperativity and, as a result, only one molecule of T_4_ is bound to TTR under physiological conditions (Ferguson et al. 1975). Due to TTR’s suggested role as a mediator for the transport of thyroid hormones across the blood–brain barrier and the placenta, structurally specific binding of PCB metabolites to T_4_ binding sites on TTR is assumed to interfere with the delivery of thyroid hormones to target tissues in the brain and the fetus and may also facilitate the transport of bound ligands to these compartments (Brouwer et al. 1998, 1999; Mortimer et al. 2012; Schreiber et al. 1995).

Structural similarities between PCB sulfates and T_4_ led us to hypothesize that PCB sulfates represent a class of high-affinity ligands for human TTR. To test this hypothesis, we characterized the binding of five LC-PCB sulfates to TTR and compared their equilibrium dissociation constants (*K*_d_ values) to those determined for their hydroxylated precursors and for T_4_. In addition, we conducted docking simulations to determine the potential molecular interactions of these sulfates within the T_4_ binding site of TTR. Additional studies addressed the reversibility of the interaction of PCB sulfates with TTR and the likelihood that human sulfotransferase 1A1 (hSULT1A1) can catalyze the formation of these PCB sulfates.

The parent PCBs of all metabolites used in this study have been detected in Chicago, Illinois, air (Hu et al. 2010). Of particular interest is the sulfate ester derived from PCB 11. Its parent PCB congener is a major component of PCBs present in Chicago and Cleveland, Ohio, air, yet it was never contained in any of the commercial PCB mixtures (Hu et al. 2010; Persoon et al. 2010). Current research suggests that PCB 11 is a by-product of paint and pigment production, thus representing a continuing source for PCB exposure (Hu and Hornbuckle 2010).

## Methods

*Chemicals*. Hydroxylated PCBs (4´-chloro-biphenyl-2-ol, 4´-chloro-biphenyl-3-ol, 4´-chloro-biphenyl-4-ol, 3,3´-dichloro-biphenyl-4-ol, and 3´,4´-dichloro-biphenyl-4-ol) and the ammonium salts of 2´-sulfooxy-4-chloro-biphenyl, 3´-sulfooxy-4-chloro-biphenyl, 4-chloro-4´-sulfooxy-biphenyl, and 3,4-dichloro-4´-sulfooxy-biphenyl were synthesized and characterized as previously described (Lehmler and Robertson 2001; Li et al. 2010). The ammonium salt of 3,3´-dichloro-4´-sulfooxy-biphenyl was synthesized and characterized as described in Supplemental Material, p. 2 (http://dx.doi.org/10.1289/ehp.1206198). 8-Anilinonaphthalene-1-sulfonic acid (ANS), l-thyroxine sodium salt pentahydrate (i.e., T_4_), adenosine-3´-phosphate-5´-phosphosulfate lithium salt hydrate (PAPS), adenosine-3´,5´-diphosphate sodium salt (PAP), sodium phosphate monobasic, and TTR purified from human plasma (> 95%) were purchased from Sigma-Aldrich (St. Louis, MO). TTR was used without further purification and its purity was routinely confirmed by SDS-PAGE. The protein concentration was determined by the Bradford assay. PAPS was purified to ≥ 98% purity according to a procedure published by Sekura (1981). Cytosolic extract of *E. coli* expressing recombinant hSULT1A1 (cytosolic protein concentration of 10 mg/mL) (Xenotech, Lenexa, KS) was used without further purification. Acetonitrile was purchased from Fisher Scientific (Hampton, NH). Sodium chloride, potassium phosphate monobasic, and ammonium chloride were obtained from Research Products International (Mt. Prospect, IL).

**Figure 1:**
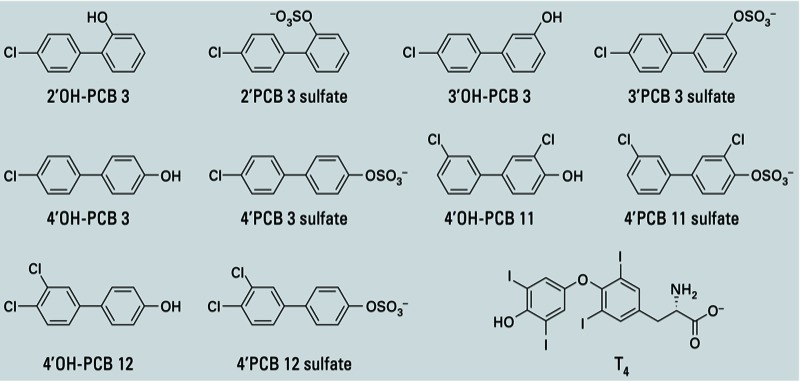
Chemical structures of PCB sulfates, OH-PCBs, and T_4_. PCB sulfates used in this study were synthesized as ammonium salts.

*ANS displacement assay*. ANS displacement studies have frequently been used to determine dissociation constants for potential ligands of TTR (Cao et al. 2010; Cheng et al. 1977; Smith et al. 1994). We determined *K*_d_ values for PCB metabolites and T_4_ utilizing a modified version of a previously published procedure (Cheng et al. 1977). A solution containing 0.5 µM TTR and 5 µM ANS (total volume 1,000 µL) in phosphate buffer [50 mM sodium phosphate, 100 mM NaCl (sodium chloride); pH 7.4] was titrated with small aliquots of PCB metabolites or T_4_ (structures shown in Figure 1) using a glass syringe (Hamilton, Reno, NV). The displacement of ANS was monitored by measuring the decrease in fluorescence intensity at 470 nm upon excitation of the molecule at 410 nm in a Spectramax M5 fluorimeter (Molecular Devices, Sunnyvale, CA) [for details, see Supplemental Material, Figure S1 (http://dx.doi.org/10.1289/ehp.1206198)]. Three fluorescence measurements were averaged per determination, and at least three separate determinations were made at each ligand concentration. The protocol was optimized for ligand concentrations of ≤ 2,000 nM, and assays were conducted at 25°C (± 0.2°C) in quartz cuvettes with a 1-cm path length. The fluorescence was corrected for dilution (≤ 4.6% of the total volume) and was found to be unaffected by the solvent of the ligands [0.5 mM NaOH (sodium hydroxide)] and the duration of the assay (see Supplemental Material, Figure S2). The total change in pH was ≤ 0.01 pH units. The concentration of ANS in phosphate buffer was determined spectrophotometrically at 350 nm using a molar extinction coefficient of 6.3 × 10^3^/cm/M (Kolb and Weber 1975). Binding data were evaluated by fitting the means of each determination to both a one-site plus nonspecific binding equation [*y* = (*B*_max1_ × *x*)/(*K*_d1_ + *x*) + **Ns**] and a two-site binding equation [*y* = (*B*_max1_ × *x*)/(*K*_d1_ + *x*) + (*B*_max2_ × *x*)/(*K*_d2_ + *x*)]. In these equations, *K*_d1_ is the dissociation constant for the TTR-ligand complex and *K*_d2_ is the dissociation constant for the TTR complex with two bound ligands, *B*_max1_ and *B*_max2_ are the relative changes in fluorescence required to saturate the respective binding sites and **Ns** is a constant representing low-affinity interactions. In this case, the **Ns** term includes both the low-affinity second T_4_ binding site in TTR and any other low-affinity interaction with the protein. The variables *x* and *y* represent ligand concentrations and changes in fluorescence (Δ fluorescence), respectively. Best fits for *K*_d1_ were obtained by fitting data in the 0–100 nM range to the one-site plus nonspecific binding equation. In order to determine *K*_d2_ values, all available data points were fit to the two-site binding equation.

*HPLC analysis of PCB sulfates*. TTR (0.5 µM) and PCB sulfate (5 µM) were incubated in phosphate buffer (50 mM sodium phosphate, 100 mM NaCl; pH 7.4) at 25°C (± 0.5°C) for 120 min. After 0, 60, and 120 min, 20 µL samples were analyzed for PCB sulfates by chromatography on a C18 HPLC column with a linear acetonitrile gradient (15–95%) in triethylammonium acetate (1% vol/vol; pH 7.5). Detailed HPLC conditions and representative chromatograms are provided in Supplemental Material, Figure S3 (http://dx.doi.org/10.1289/ehp.1206198).

*Molecular docking simulations*. All docking simulations were performed using SYBYL X software (Tripos, St. Louis, MO). From the Protein Data Bank (PDB; http://www.wwpdb.org/), we selected the crystal structure complex of TTR with bound 2,´6´-difluorobiphenyl-4-carboxylic acid (PDB no. 2F7I) to constitute the receptor model for human TTR. The receptor structure was prepared according to protocols provided by Tripos. Briefly, protein chain termini were set into their charged states, and hydrogen atoms were added before staged minimizations were performed with the Powell method, using the MMFF94s force field. Ligands present in the crystal structure were extracted from the receptor and used to define the active site (protomol). Default settings were used for protomol generation. Ligands (2´PCB 3 sulfate, 3´PCB 3 sulfate, 4´PCB 3 sulfate, 4´PCB 11 sulfate, 4´PCB 12 sulfate) were modeled in ChemBioDraw 12.0 (PerkinElmer, Waltham, MA) and imported into a SYBYL X database. All ligands were energy-minimized using the Tripos force field and docked into prepared receptor structures using the Surflex-Dock module in Geom mode. Twenty different binding poses were generated per molecule and ranked according to their binding energies. Images were created using the PyMOL Molecular Graphics System (version 1.5.0.4; Schrödinger LLC, New York, NY).

*Sulfotransferase assay*. The assays for sulfation of OH-PCBs catalyzed by hSULT1A1 were conducted according to a previously published procedure (Liu et al. 2009). Assay mixtures containing 0, 10, 20, or 30 µM of the potential substrate were preincubated at 37°C in the presence of 200 µM PAPS and 7.5 mM 2-mercaptoethanol in 250 mM potassium phosphate at pH 7.0. After adding 1 µL of cytosolic extract of *E. coli* expressing hSULT1A1 (10 µg cytosolic protein), the reaction mixture (total volume 30 µL) was incubated for 10 min before the reaction was terminated by the addition of 30 µL methanol. Samples (20 µL) were subsequently analyzed by HPLC to determine the amount of PAP formed in the reaction (Sheng et al. 2001).

## Results

**Figure 2:**
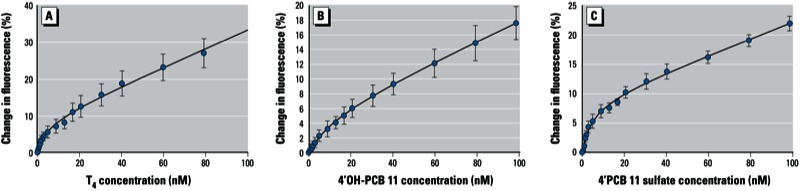
Binding of T_4_, 4´OH-PCB11, and 4´PCB 11 sulfate to the high-affinity site on TTR. Data for the binding of T_4_ (*A*), 4OH-PCB 11 (*B*), and 4´PCB 11 sulfate (*C*) were determined by ANS displacement using ligand concentrations between 0 and 100 nM. Data were fit to a one-site plus nonspecific binding equation; data points represent means of at least three experiments (Table 1); error bars indicate SDs.

*Binding of T_4_ and PCB metabolites to TTR*. We determined *K*_d_ values for T_4_ of 4.7 ± 1.1 nM and 234 ± 11 nM at the high- and low-affinity binding sites on TTR, respectively [Figure 2A; see also Supplemental Material, Figure S4A (http://dx.doi.org/10.1289/ehp.1206198)]. These values are in agreement with previously published data for T_4_ binding to TTR (Cheng et al. 1977; Smith et al. 1994; Ucan-Marin et al. 2009, 2010). After validating the assay conditions, we determined *K*_d_ values for five PCB sulfates and their hydroxylated precursors (Figure 2; see also Supplemental Material, Figure S5). All five PCB sulfates were found to be ligands for the high-affinity binding site, with *K*_d1_ values ranging from 4.8 nM to 16.8 nM. 2´PCB 3 sulfate, 3´PCB 3 sulfate, and 4´PCB 11 sulfate exhibited significantly higher affinities (i.e., lower *K*_d1_ values) than their respective OH-PCBs (Table 1). Although the *K*_d1_ for 4´PCB 3 sulfate (14.5 nM) was lower than the one for 4´OH-PCB 3 (19.3 nM), the difference was not significant. The lowest *K*_d1_ was obtained for 4´PCB 11 sulfate (4.8 nM) (Figure 2C and Table 1). 4´OH-PCB 12 had the highest-affinity binding in this study, and it exhibited a significantly lower dissociation constant (*K*_d1_ = 2.7 nM) than its sulfate (16.8 nM) and T_4_ (4.7 nM). *K*_d_ values determined for the low-affinity binding site revealed a clustered pattern for mono- and dichlorinated PCB metabolites. Although dichlorinated PCB metabolites bound with *K*_d2_ values ranged from 624 to 825 nM (Table 1; see also Supplemental Material, Figure S4A), the chosen concentration range of ≤ 2,000 nM was insufficient to facilitate quantitative displacement of ANS from the second binding site by PCB 3 metabolites (see Supplemental Material, Figure S4B). All lower chlorinated PCB metabolites exhibited significantly higher *K*_d2_ values than T_4_.

**Table 1:** *K*_d_ values determined for PCB metabolites and T_4_.

Compound	*n*	*K*_d1_ (nM)	*p*-Value^*a*^	*p*-Value^*b*^	*K*_d2_ (nM)	*p*-Value^*c*^
T_4_	4	4.7 ± 1.1	–	–	234 ± 11	–
2´OH-PCB 3	4	19.8 ± 6.0	–	0.004	^*d*^	–
2´PCB 3 sulfate	3	5.4 ± 1.2	0.015	0.458	^*d*^	–
3´OH-PCB 3	4	14.8 ± 2.0	–	< 0.001	^*d*^	–
3´PCB 3 sulfate	3	8.2 ± 1.0	0.007	0.008	^*d*^	–
4´OH-PCB 3	3	19.3 ± 3.8	–	< 0.001	^*d*^	–
4´PCB 3 sulfate	4	14.5 ± 4.0	0.206	0.005	^*d*^	–
4´OH-PCB 11	8	13.9 ± 1.8	–	< 0.001	624 ± 207^*e*^	0.004
4´PCB 11 sulfate	3	4.8 ± 0.7	< 0.001	0.897	624 ± 63	< 0.001
4´OH-PCB 12	3	2.7 ± 0.5	–	0.035	825 ± 47	< 0.001
4´PCB 12 sulfate	4	16.8 ± 5.8	0.014	0.008	742 ± 58	< 0.001
^***a***^PCB sulfates compared with their OH-PCBs (*K*_d1_).^***b***^PCB sulfates compared with T_4_ (*K*_d1_).^***c***^PCB sulfates compared with T_4_ (*K*_d2_).^***d***^*K*_d_ values could not be determined for the second site because of low-affinity binding.^***e***^Best fit was obtained using a one-site plus nonspecific binding equation.						

*Stability of PCB sulfates in the assay.* In order to determine the stability of PCB sulfates under the conditions of the assay for binding to TTR, we developed an HPLC procedure for the separation and quantification of PCB sulfates and their respective OH-PCBs. Using a linear 15–95% acetonitrile gradient, we were able to separate all five matching pairs of PCB sulfates from their OH-PCB precursors [see Supplemental Material, Figure S3 (http://dx.doi.org/10.1289/ehp.1206198)]. Using this procedure, we quantitatively recovered 5 µM samples of 2´PCB 3 sulfate, 3´PCB 3 sulfate, 4´PCB 3 sulfate, 4´PCB 11 sulfate, and 4´PCB 12 after incubation with 0.5 µM TTR under ANS assay conditions (see Supplemental Material, Figure S6). Moreover, quantitative recovery of the PCB sulfates by HPLC indicated that the binding to TTR is reversible under the conditions of HPLC analysis.

**Figure 3:**
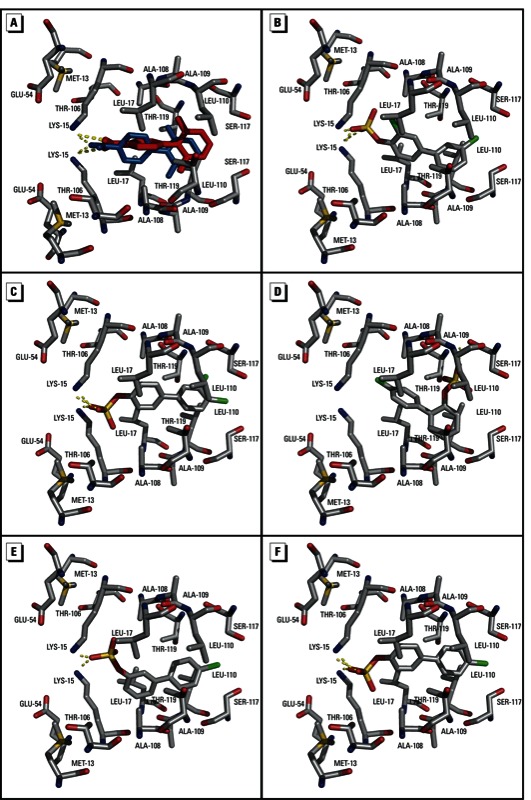
Molecular docking of PCB sulfates into the T_4_ binding site on TTR. (*A*) Comparison between the original crystal structure (PDB no. 2F7I, blue) and docked 2´,6´-difluorobiphenyl-4-carboxylic acid/TTR (red) complex. (*B–F*) Views of the TTR binding site are shown after docking with 4´PCB 11 sulfate (*B*), 4´PCB 12 sulfate (*C*), 2´PCB 3 sulfate (*D*), 3´PCB 3 sulfate (*E*), and 4´PCB 3 sulfate (*F*).

*Selection of a receptor structure for docking simulations*. TTR is a 55-kDa homotetrameric protein consisting of a dimer of dimers in a twofold symmetrical arrangement. The interface between the two dimers forms a channel that contains two T_4_ binding sites (Blake and Oatley 1977; Wojtczak et al. 1996). Each T_4_ binding site is lined with mostly hydrophobic residues that form two symmetrical sets of three halogen-binding pockets, P1–P3 and P1´–P3´ (Blake and Oatley 1977). P1 and P1´ are located on the outside of the channel and comprise side chains of residues Met13, Lys15, Thr106, and Ala108. On the outermost part of this pocket, Lys15 and Glu54 form a charged region that provides the possibility for polar interactions between protein and ligands. The side chains of Lys15, Leu17, Ala109, and Leu110 form P2 and P2´. P3 and P3´, the innermost binding pockets, are formed by side chains of Ala108, Leu110, Ser117, and Thr119. The crystal structure of T_4_ bound to TTR (PDB no. 2ROX) indicates a binding orientation in which the alanyl moiety of T_4_ facilitates hydrogen bonding interactions with Lys15 and Glu54, and the phenol is oriented towards Ser117 and Thr119 (Wojtczak et al. 1996). Iodine substituents in the 3 and 5 position of thyroxine are positioned in P2 and P3, respectively. We initially selected three TTR crystal structure complexes with different bound ligands (PDB no. 2ROX, 2G9K, and 2F7I) from the PDB to constitute the receptor model for TTR. Following extraction of the ligands and subsequent docking of the extracted ligands into the generated protomol, the closest match was obtained for the complex of 2´,6´-difluorobiphenyl-4-carboxylic acid and TTR (PDB no. 2F7I). The docked ligand revealed similar positioning and molecular interactions within the binding site as compared with the crystal structure (Figure 3A). In particular, the carboxylate of 2´,6´-difluorobiphenyl-4-carboxylic acid engages in hydrogen bonding interactions with Lys15. The 2´ and 6´ fluorine substituents are located in P3 and P3´. Because of the lack of fluorines on the unsubstituted ring, it shows some flexibility and is twisted approximately 90° in the docked structure as compared with the crystal structure.

*Docking simulations for PCB sulfates*. Using PDB no. 2F7I as the receptor model, we were able to dock all five PCB sulfates into the T_4_ binding site (Figure 3B–F). To be consistent in the interpretation of our docking results, only the lowest-energy binding conformation is indicated per PCB sulfate. The lowest-energy binding conformations indicated an orientation that enables hydrogen bonding interactions between the sulfate groups and Lys15. The only exception was 2´PCB 3 sulfate, where hydrogen bonding with Lys15 residues was sterically unfavorable. Instead, the sulfate moiety of 2´PCB 3 sulfate formed a hydrogen bond with Leu110, resulting in a reverse orientation of this metabolite. The binding modes of 4´PCB 11 sulfate and 4´PCB 12 sulfate, the only two compounds with chlorines in the *meta* position were similar to those previously determined for T_4_. Whereas the sulfate group facilitates hydrogen bonding with Lys15, the *meta*-positioned chlorines appeared to be inserted, analogously to the iodines in T_4_, into P2 and P3. It should be noted that some of the higher-energy binding poses obtained for PCB sulfates exhibited an antiparallel orientation with the sulfate group pointing towards the central Ser117.

**Figure 4:**
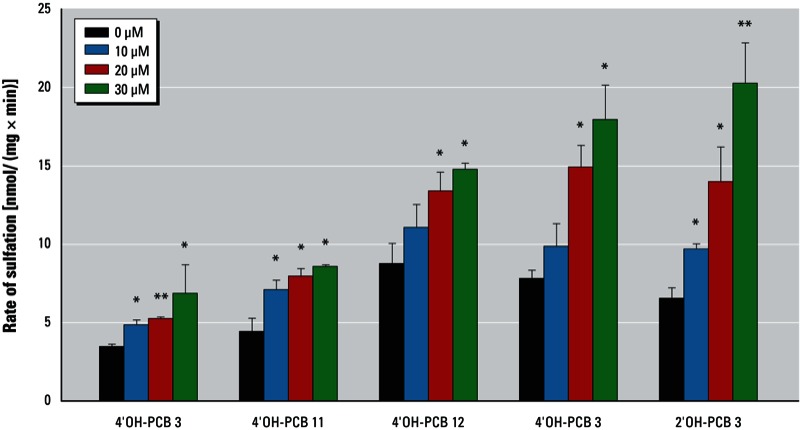
OH-PCBs are substrates for human SULT1A1. The sulfation of 2´OH-PCB 3, 3´OH-PCB 3, 4´OH-PCB 3, 4´OH-PCB 11, and 4´OH-PCB 12 is catalyzed by a cytosolic extract of *E. coli* expressing recombinant hSULT1A1. *p < 0.05, and **p < 0.001 compared with 0 µM OH-PCB

*OH-PCBs as substrates for hSULT1A1*. Because formation of PCB sulfates requires their metabolic formation from OH-PCBs, we determined the rate of sulfation of OH-PCBs by recombinant hSULT1A1. The five OH-PCBs that were tested in this study were substrates for the enzyme and exhibited a concentration-dependent increase in the rate of sulfation (Figure 4). The highest specific activities were determined for the *ortho*-hydroxylated 2´OH-PCB 3 and for the *meta*-hydroxylated 3´OH-PCB 3. The *para*-hydroxylated 4´OH-PCB 3, 4´OH-PCB 11, and 4´OH-PCB 12 exhibited slightly lower specific activities. Thus, the hSULT1A1, a major human cytosolic sulfotransferase, catalyzes the sulfation of these OH-PCBs.

## Discussion

Several recent studies have demonstrated the potential of OH-PCB metabolites to serve as substrates for sulfotransferases *in vitro* and *in vivo* (Dhakal et al. 2012; Ekuase et al. 2011; Liu et al. 2009). Importantly, using a rat model Dhakal et al. (2012) observed that serum concentrations of PCB 3 sulfates greatly exceeded those of their respective OH-PCBs, thereby suggesting that in contrast to the higher-chlorinated PCBs and OH-PCBs that tend to be retained in blood and adipose tissue, LC-PCBs undergo relatively rapid oxidation and conjugation upon absorption. Considering the abundance of LC-PCBs in indoor and outdoor air samples (Hu et al. 2010; Ludewig et al. 2008), these discoveries raise concern about both the fate and potential toxicities of PCB sulfates.

In the present study, we have demonstrated the ability of five LC-PCB sulfates to bind with high affinity to the most physiologically relevant binding site for T_4_ on human TTR. Using a fluorescence displacement assay, we determined that *K*_d_ values for the high-affinity binding site ranged from 4.8 nM to 16.8 nM and were thus comparable to T_4_ (4.7 nM). Importantly, four of the five PCB sulfates (2´PCB 3 sulfate, 3´PCB 3 sulfate, 4´PCB 3 sulfate, and 4´PCB 11 sulfate) interacted with higher affinities than their corresponding OH-PCBs. Although OH-PCBs have been previously shown to be high-affinity ligands for TTR that are capable of competing with T_4_ (Gutleb et al. 2010; Lans et al. 1993; Rickenbacher et al. 1986), our results now establish the potential for binding of PCB sulfates to TTR. Some of the higher-chlorinated OH-PCBs are several times more potent ligands for TTR than T_4_ (Chauhan et al. 2000; Rickenbacher et al. 1986; Ucan-Marin et al. 2009, 2010). It is possible that some higher-chlorinated PCB sulfates may exhibit even higher affinities for TTR than the lower-chlorinated ones used here, although testing of this hypothesis and evaluating the role of sulfation in the metabolism of such higher-chlorinated PCBs will await further studies. Besides PCB mono-sulfates, which constituted the largest group of metabolites in serum of PCB 3–exposed rats, the recent metabolism study on PCB 3 also reported detectable levels of mercapturic acid conjugates and sulfate esters derived from dihydroxylated metabolites, albeit at lower levels (Dhakal et al. 2012). The binding characteristics of such metabolites remain to be characterized in subsequent studies because they might contribute to the displacement of T_4_ from binding sites on TTR. Although glucuronide conjugates were not major metabolic products for PCB 3 (Dhakal et al. 2012), we also cannot rule out the possibility of biological effects of these conjugates.

Supporting evidence for high-affinity binding of PCB sulfates to TTR was provided by molecular docking simulations. With the exception of 2´PCB 3 sulfate, the sulfate moiety facilitated hydrogen bonding interactions with Lys15 residues, thereby dictating the binding orientation of the PCB sulfate. The *ortho*-sulfate group in 2´PCB 3 sulfate appears to sterically prevent this interaction. Instead, the orientation of the molecule is reversed, which enables hydrogen bonding of the sulfate with Leu110. Among the PCB 3 sulfates, 2´PCB 3 sulfate bound significantly better to the high-affinity binding site than 3´PCB 3 sulfate or 4´PCB 3 sulfate (Table 1), and this may be due to the alternate binding orientation within the T_4_ binding site. In addition, the observed increased affinity of 4´PCB 11 sulfate as compared with 4´PCB 12 sulfate can be explained by the presence of a second *meta*-chlorine that provides the opportunity for additional anchorage within binding pockets P3 or P3´. This finding is consistent with previous reports indicating that *meta*- and *para*-chlorination increases affinities of OH-PCBs to TTR (Chauhan et al. 2000; McKinney et al. 1987; Rickenbacher et al. 1986).

Similar to T_4_, *K*_d_ values determined for the second binding site on TTR were found to be at least two orders of magnitude higher than those determined for the high-affinity binding site (Cheng et al. 1977; Smith et al. 1994). *K*_d2_ values determined for all PCB metabolites were significantly higher than for T_4_. These weaker interactions with the second binding site are consistent with the negative cooperativity between these sites that is observed with T_4_ (Ferguson et al. 1975). T_4_, although structurally similar to PCB metabolites, is a larger molecule with a higher degree of halogenation than the PCB metabolites used in this study. These differences may account for a sterically more favorable binding to the second site. However, under physiological conditions, concentrations of T_4_ are too low to allow binding to the low-affinity binding site (Liz et al. 2010). Consequently, the displacement of T_4_ by PCB metabolites primarily affects the high-affinity binding site *in vivo*, whereas both sites may have relevance in the (inter-tissue) transport and retention of xenobiotics.

Whereas in cerebrospinal fluid TTR is the only TH transporter, in human serum the displacement of T_4_ from binding sites on two additional T_4_ transport proteins, albumin and thyroxine binding globulin, may be a contributing factor in the saturation of T_4_ binding sites and should be addressed in further studies. However, previous research has emphasized the displacement of T_4_ from TTR as a key contributing mechanism in organohalogen-induced hypothyroxinemia (Gutleb et al. 2010; Kodavanti and Curras-Collazo 2010). TTR has additional relevance owing to its function as a mediator for the transport of thyroid hormones across the placenta and the blood–brain barrier, and it has been suggested that the binding of PCB metabolites to TTR may facilitate their transport to the cerebrospinal fluid and to the fetus (Brouwer et al. 1998, 1999; Schreiber et al. 1995; Southwell et al. 1993). Recently, it was reported that TTR has the potential for translocation across the placenta into the fetal circulation and represents a potential shuttle system for thyroid hormones and for exogenous compounds (Mortimer et al. 2012). Considering their ability to bind with high affinity to TTR, the PCB sulfates used in this study possess the basic requirements for such inter-tissue transport. Moreover, the observations that their binding affinities do not exceed those determined for T_4_, and that their interactions are noncovalent, points towards their ability to dissociate from TTR upon delivery to potential target tissues. Although higher-chlorinated PCB sulfates remain to be examined for their binding to TTR, previous reports suggest that the higher-chlorinated OH-PCBs may be less able to dissociate and may be retained bound to TTR in the circulation (Bergman et al. 1994; Brouwer et al. 1998). Likewise, future research will be required to further assess levels of human exposure to PCB sulfates as well as the *in vivo* effects of PCB sulfates on T_4_ concentrations in susceptible tissues and/or transport of PCB sulfates into those tissues.

## Conclusions

We have identified five LC-PCB sulfates as high-affinity ligands for human TTR. Notably, their binding affinities were similar to those determined for their respective OH-PCB precursors and for T_4_. The binding interactions between all five PCB sulfates and TTR were found to be noncovalent. We used docking simulations to calculate their lowest-energy binding conformations within the thyroxine binding sites of human TTR, thereby providing corroborating evidence for their high-affinity binding, and insight into potential binding orientations. Moreover, the corresponding OH-PCBs were found to be substrates for hSULT1A1, a major human cytosolic sulfotransferase. Thus, these results indicate, for the first time, a potential toxicological relevance of PCB sulfates in the disruption of thyroid hormone homeostasis in tissues dependent upon TTR-mediated transport.

## Supplemental Material

(1.5 MB) PDF
