# Supplementary material for: Sulfated Metabolites of Polychlorinated Biphenyls Are High-Affinity Ligands for the Thyroid Hormone Transport Protein Transthyretin
Source: Environ Health Perspect. 2013 Apr 12;121(6):657–62. doi: 10.1289/ehp.1206198 (PMC3672920; doi:10.1289/ehp.1206198)
Supplement: (1.5 MB) PDF [file ehp.1206198.s001.pdf]

Supplemental Material

Sulfated Metabolites of Polychlorinated Biphenyls Are High-Affinity Ligands for the Thyroid Hormone Transport Protein Transthyretin

Fabian A. Grimm[1,2], Hans-Joachim Lehmler[1,3], Xianran He[3], Larry W. Robertson[1,3], and Michael W. Duffel[1,2]

[1] Interdisciplinary Graduate Program in Human Toxicology, The University of Iowa, Iowa City, Iowa, USA

[2] Department of Pharmaceutical Sciences and Experimental Therapeutics, College of Pharmacy, The University of Iowa, Iowa City, Iowa, USA

[3] Department of Occupational and Environmental Health, College of Public Health, The University of Iowa, The University of Iowa, Iowa City, Iowa, USA

Table of Contents:

Synthesis and Characterization of 4’PCB 11 sulfate .... pg 2

Supplemental Material, Figure S1 .... pg 3

Supplemental Material, Figure S2 .... pg 4

Supplemental Material, Figure S3 .... pg 5

Supplemental Material, Figure S4 .... pg 6

Supplemental Material, Figure S5 .... pg 7

Supplemental Material, Figure S6 .... pg 8

References .... pg 9

Synthesis and Characterization of 4'PCB 11 sulfate

The ammonium salt of 3,3'-dichloro-4'-sulfooxy-biphenyl was synthesized in two steps from 3,3'-dichloro-biphenyl-4-ol. Briefly, 3,3'-dichloro-biphenyl-4-ol was converted into the corresponding 2,2,2-trichloroethyl-protected sulfuric acid diester, which was subsequently deprotected with Zn/HCO_2NH_4 in methanol to yield the desired ammonium salt after column chromatography on silica gel with $CH_2Cl_2/CH_3OH/NH_4OH$ (15:5:0.5, v/v).(Li et al. 2010)

3,3'-Dichlorobiphenyl-4-yl 2,2,2-trichloroethyl sulfate

Colorless oil. Yield = 93 %. ^{1}H NMR (400 MHz, $CDCl_3$): δ/ppm 4.96 (s, 2H, CH_2), 7.36-7.40 (m, 3H), 7.49 (dd, 1H, *J* = 2.0 Hz, *J* = 8.4 Hz), 7.50-7.51 (m, 1H), 7.59 (d, 1H, *J* = 8.4 Hz), 7.66 (d, 1H, *J* = 2.0 Hz). ^{13}C NMR (100 MHz, $CDCl_3$): δ/ppm 80.9, 92.3, 123.4, 125.4, 127.0, 127.2, 127.4, 128.6, 129.7, 130.5, 135.2, 140.2, 140.9, 145.5. EI-MS *m/z* (relative intensity, %): 448 (22, $C_{14}H_9Cl_5O_4S^{\bullet+}$), 318 (14), 237 (100), 209 (42), 173 (23), 139 (63).

3,3'-Dichloro-4'-sulfooxy-biphenyl, ammonium salt

White solid. Yield = 88 %. mp: 220 ºC (dec.); ^{1}H NMR (400 MHz, $CDCl_3$): δ/ppm 7.35 (ddd, 1H, *J* = 0.8 Hz, *J* = 2.0 Hz, *J* = 7.8 Hz), 7.42 (pseudo t, 1H, *J* ~ 8 Hz), 7.50-7.55 (m, 2H), 7.61 (pseudo t, 1H, *J* ~ 2 Hz), 7.67 (d, 1H, *J* = 2.4 Hz), 7.70 (d, 1H, *J* = 8.4 Hz). ^{13}C NMR (100 MHz, $CDCl_3$): δ/ppm 124.1, 126.3, 127.2, 127.8, 128.2, 128.7, 129.5, 131.5, 135.9, 138.4, 142.6, 149.9. HRMS (ESI, negative): $[M]^-$ found *m/z* 316.9448, calculated for $C_{12}H_7(35)Cl_2O_4S$ 316.9448.

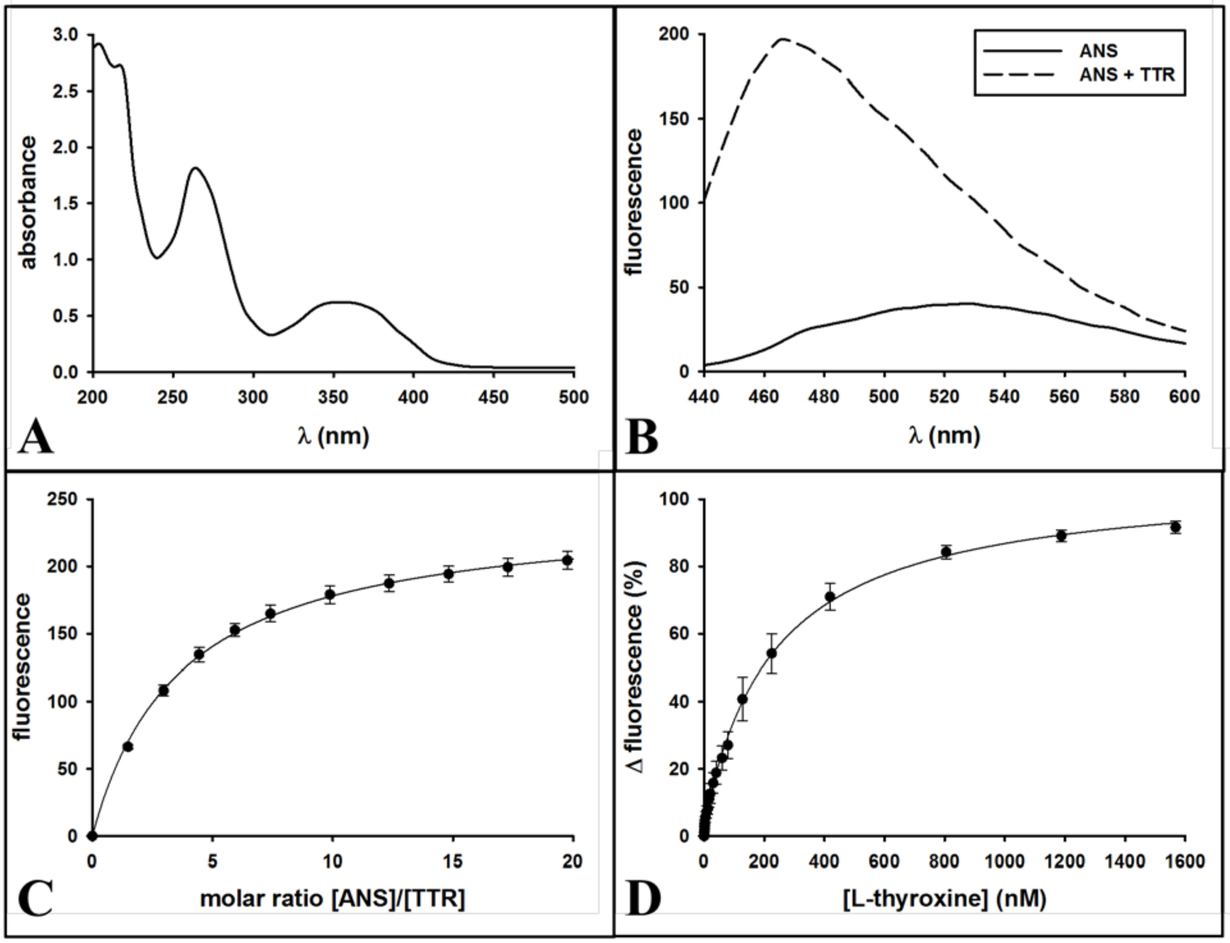

Supplemental Material, Figure S1: Determination of the binding of L-thyroxine to TTR by ANS displacement. (A) Absorbance spectrum of 100 µM ANS in phosphate buffer (50 mM sodium phosphate, 100 mM NaCl, pH 7.4); **(B)** Emission spectra of 100 µM ANS in the presence and absence of 0.5 µM TTR (exc. λ = 410 nm). When free in aqueous solution, ANS has an emission maximum at 525 nm. This maximum shifts to 465 nm, accompanied by an increase in the quantum yield, upon bi nding of ANS to TTR (Cheng et al. 1977). The displacement of ANS from TTR by a competing ligand will result in a decrease in the fluorescence signal. **(C)** In order to determine the best molar ratio of ANS and TTR to be used in the assay, we titrated a solution containing 0.5 µM TTR with aliquots of ANS. Standard deviations (n=3) are indicated in the plot. The equilibrium dissociation constant obtained for ANS was 3.7 ± 0.1 µM and was in agreement with previously published values (Cao et al. 2010; Lima et al. 2010). The binding curve indicated saturation binding up to a molar ratio of approximately 10:1 (ANS/TTR), followed by a slight, linear increase in fluorescence. **(D)** Fluorescence displacement curve for L-thyroxine. Standard deviations are indicated for all datapoints (n=4). The change in fluorescence was fit to a two-site binding equation.

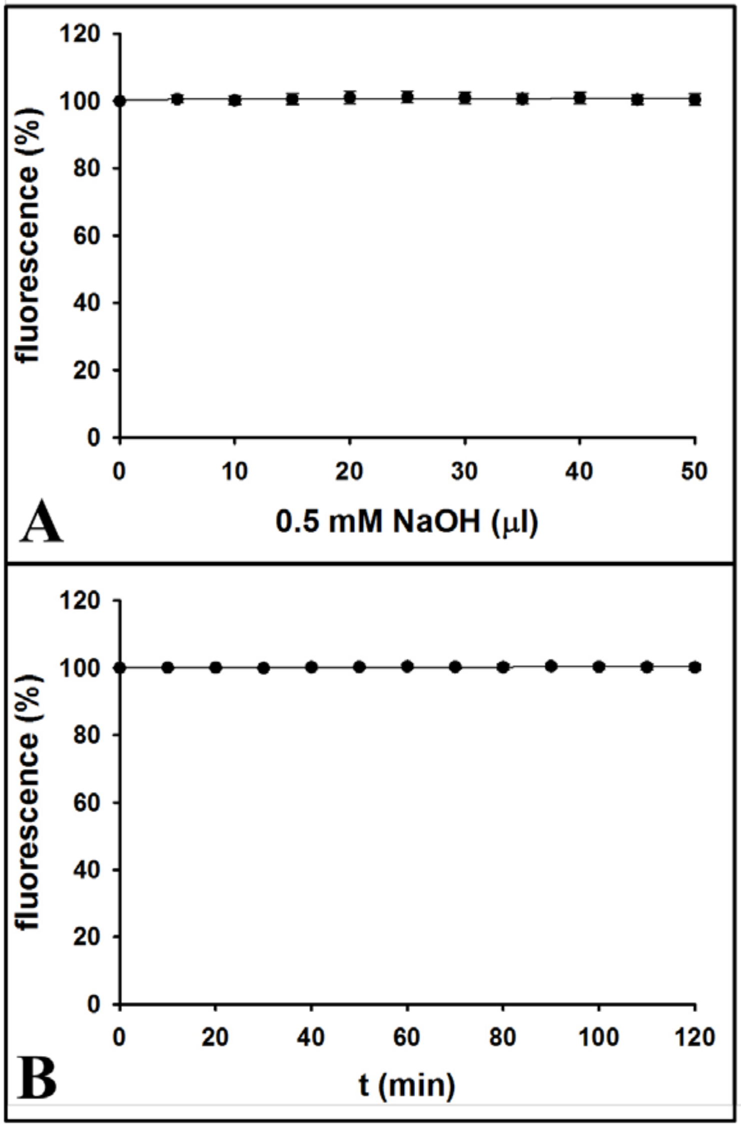

Supplemental Material, Figure S2: Solvent and time effects on ANS fluorescence.
(A) The effect of 0.5 mM NaOH, the solvent for T4 and PCB metabolites, on A NS fluorescence (exc. λ = 410 nm; em. λ = 470 nm) was assessed by titration of a solution containing 0.5 µM TTR and 5 µ M ANS with 5 µ l aliquots of 0.5 m M NaOH. Standard deviations for three individual determinations are indicated (n=3 experiments). **(B)** The effect of time on ANS fluorescence (exc. λ = 410 nm; em. λ = 470 nm) was determined by incubating 0.5 µM TTR and 5 µM ANS for 120 minutes at 25˚C.

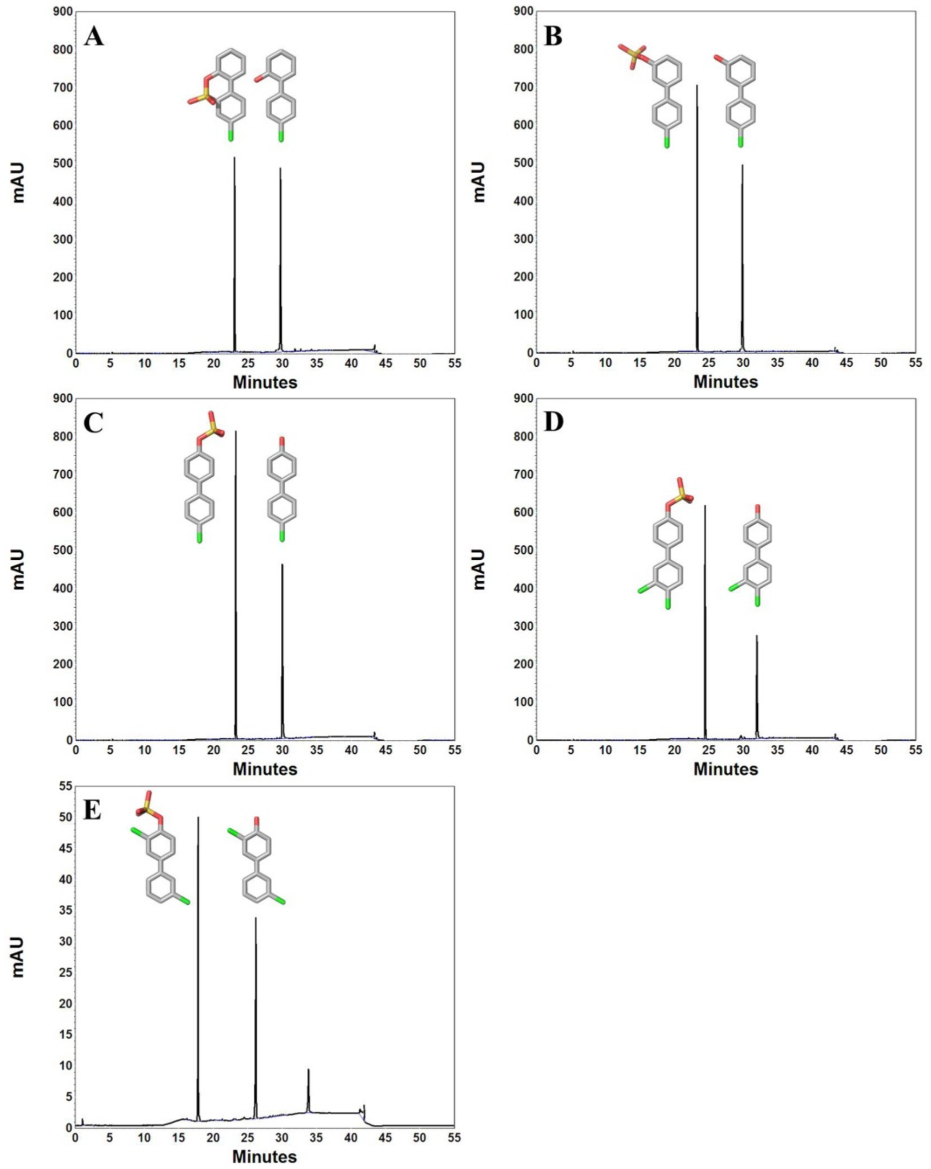

Supplemental Material, Figure S3: Representative chromatograms for the separation of OHPCBs and PCB sulfates by HPLC. Chromatograms represent the separation of 100 µM samples of 2'PCB 3 sulfate **(A)**, 3'PCB 3 sulfate **(B)**, 4'PCB 3 sulfate **(C),** 4'PCB 12 sulfate **(D)** and a 20 µM sample of 4'PCB 11 sulfate **(E)** from their OHPCB precursors. All analyses were conducted using a Shimadzu Model LC-20-AT liquid chromatograph and Model SPD-20-AT UV/VIS detector utilizing a C18 AQ 5 µm (4.6 x 250 mm) HPLC column (Grace, Deerfield, IL). PCB sulfates were separated from TTR and their corresponding OHPCBs with a linear acetonitrile gradient (0-10 min: 15 %; 10-30 min: 15% - 95%; 30-50 min: 95%; 50-55 min: 15%) in triethylammonium acetate (1 % v/v, pH 7.5) at a flow rate of 1.5 ml/min. For the separation of 4'PCB 11 sulfate and 4'OHPCB 11, the gradient was slightly modified: 0-10 min: 15 %; 10-30 min: 15% - 95%; 30-40 min: 95%; 40-55 min: 15%. Analytes were detected by measuring their absorbance at 254 nm and their concentrations were calculated by fitting their peak area integrations to a standard curve of known concentrations.

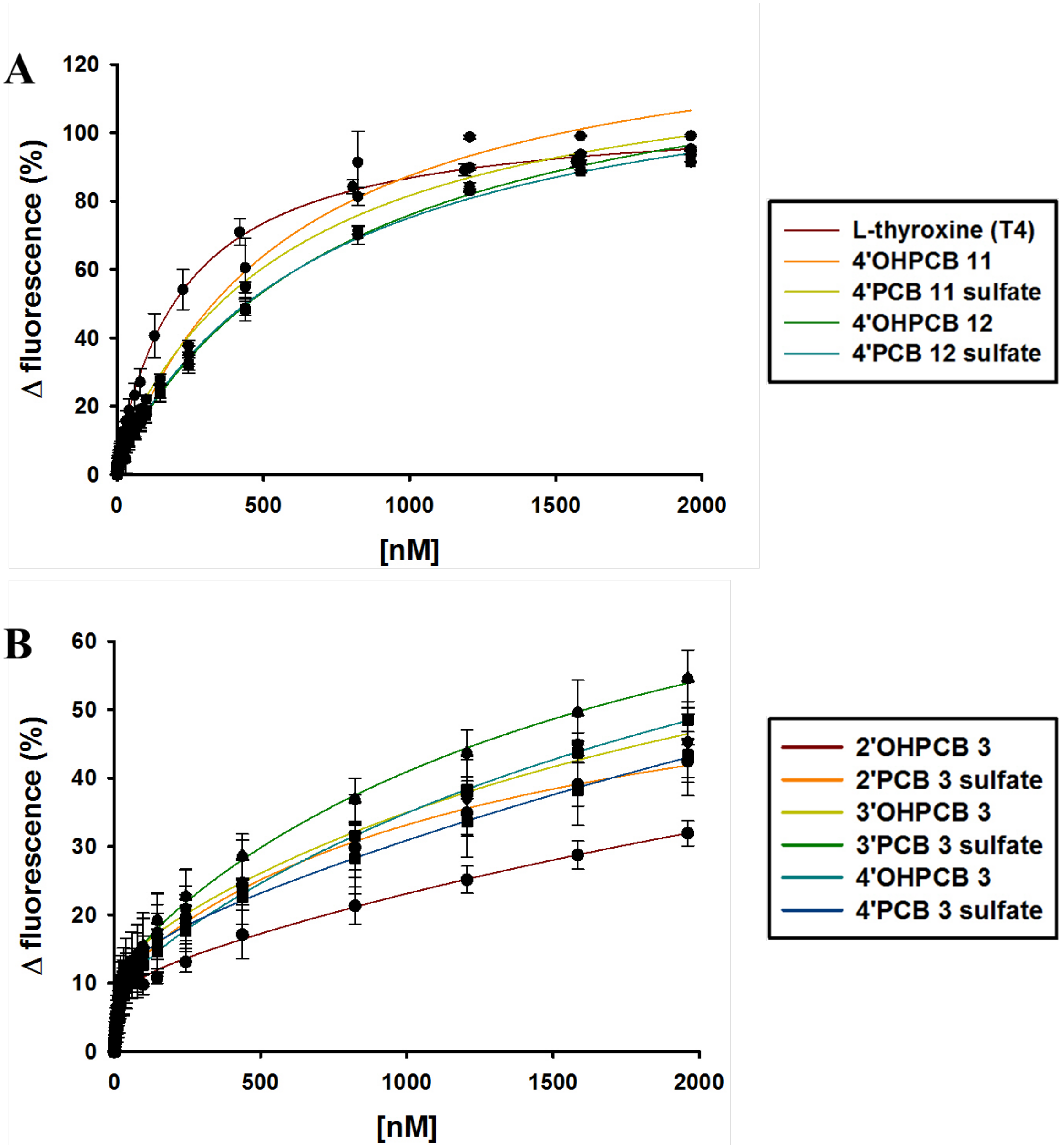

Supplemental Material, Figure S4: Binding of L-thyroxine and PCB metabolites incorporating both high-affinity and low-affinity sites. Binding curves for dichlorinated PCB metabolites and T4 **(A)** and monochlorinated PCB 3 m etabolites **(B)** as determined by ANS displacement. Data were fit to a two-site binding equation. Data points represent mean values of at least three individual experiments (Table 1). Error bars indicate standard deviations for all data points.

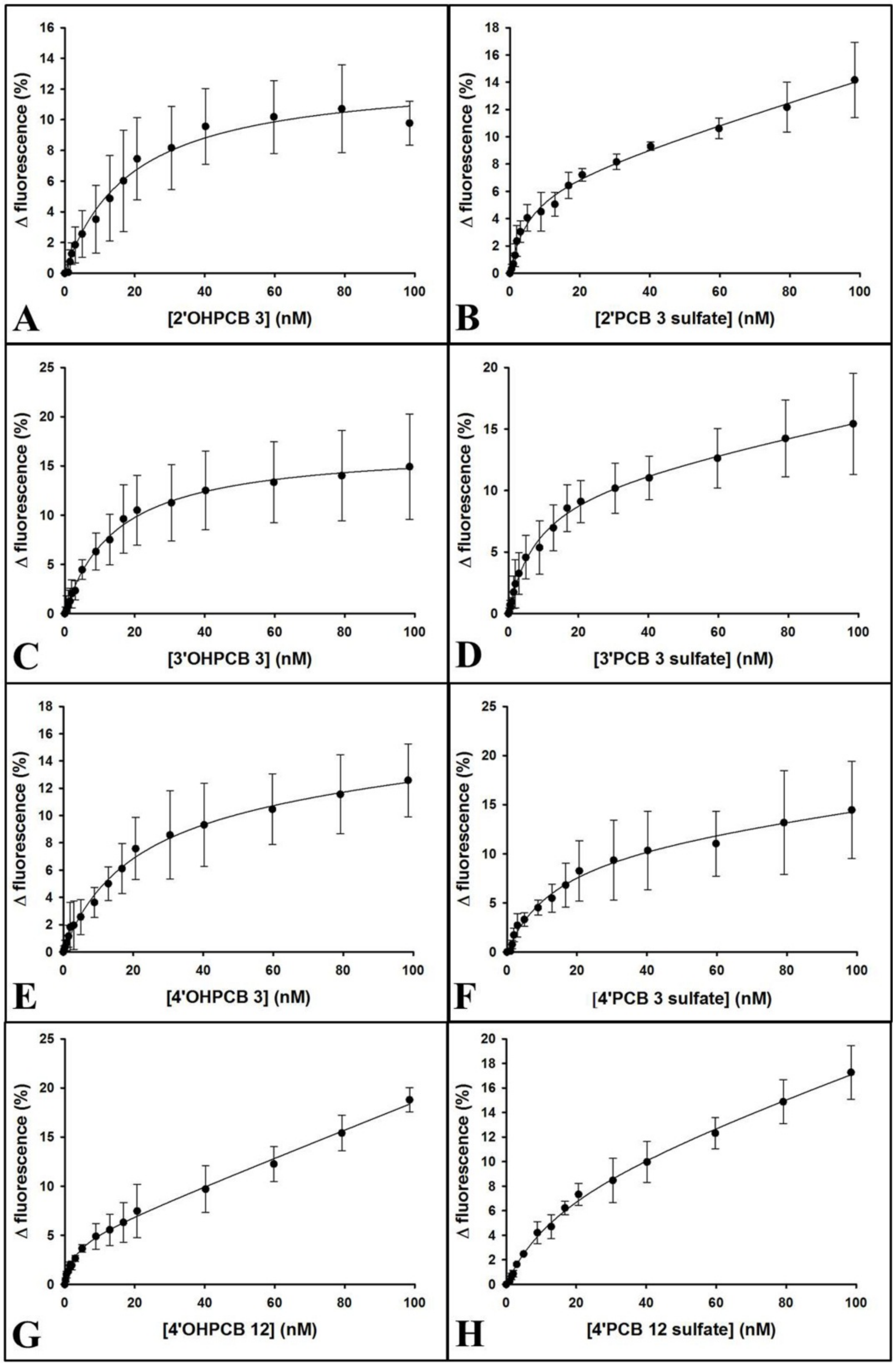

Supplemental Material, Figure S5: Determination of the binding of PCB metabolites to TTR. Binding was determined by displacement of ANS to form a complex of human TTR with 2’OHPCB 3 **(A)**, 2’PCB 3 sulfate **(B)**, 3’OHPCB 3 **(C)**, 3’PCB 3 sulfate **(D)**, 4’OHPCB 3 **(E)**, 4’PCB 3 sulfate **(F)**, 4’OHPCB 12 **(G)**, and 4’PCB 12 sulfate **(H)**. Binding curves were obtained by fitting data points between 0 and 100 nM to a one site plus nonspecific binding equation. Data points represent the means of at least three experiments (see Table 1). Error bars indicate standard deviations for each data point.

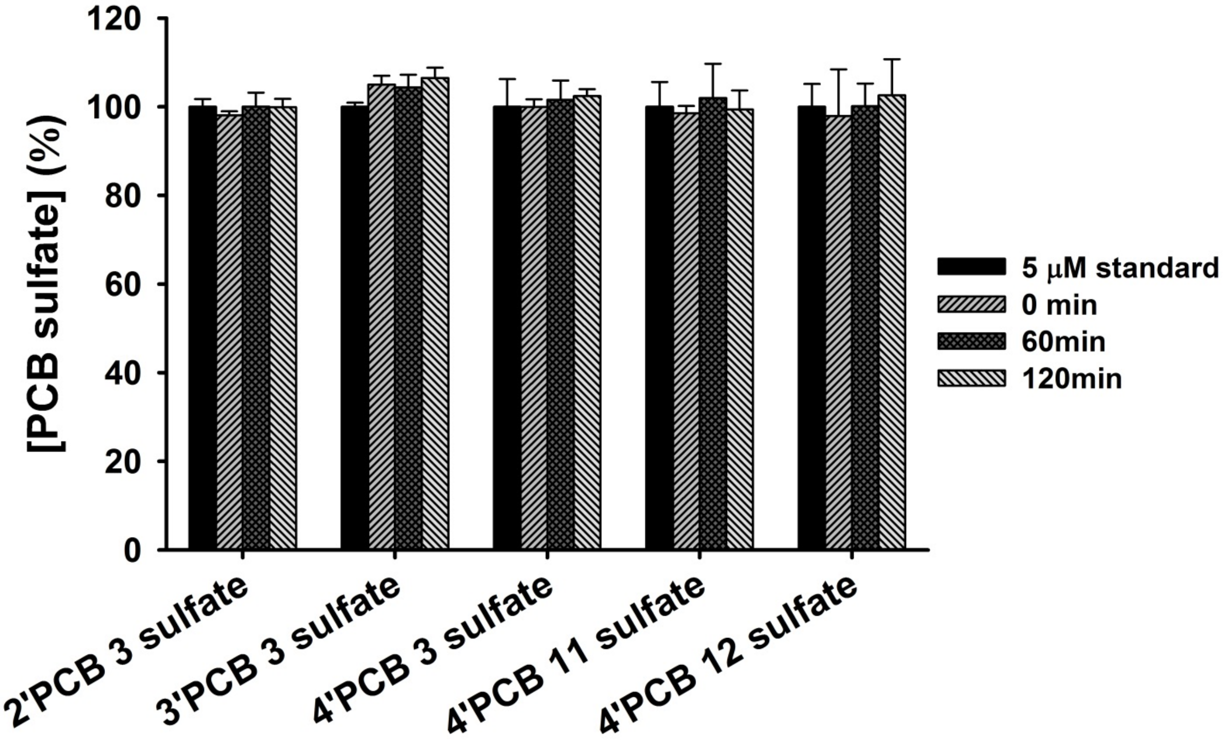

Supplemental Material, Figure S6: PCB sulfates are stable upon incubation with TTR, and the interaction is non-covalent. 5μM samples of PCB sulfates were incubated for up to 120 minutes in the presence of 0.5 μM TTR under ANS assay conditions in phosphate buffer (50 mM sodium phosphate, 100 mM NaCl, pH 7.4) at 25˚C. Their quantitative recovery (as compared to a corresponding 5 μM PCB sulfate standard) by HPLC indicated non-covalent binding interactions between PCB sulfates and TTR.

References:

Cao J, Lin Y, Guo LH, Zhang AQ, Wei Y, Yang Y. 2010. Structure-based investigation on the binding interaction of hydroxylated polybrominated diphenyl ethers with thyroxine transport proteins. Toxicology 277(1-3): 20-28.

Cheng SY, Pages RA, Saroff HA, Edelhoch H, Robbins J. 1977. Analysis of thyroid hormone binding to human serum prealbumin by 8-anilinonaphthalene-1-sulfonate fluorescence. Biochemistry 16(16): 3707-3713.

Li X, Parkin S, Duffel MW, Robertson LW, Lehmler H-J. 2010. An efficient approach to sulfate metabolites of polychlorinated biphenyls. Environ Int 36: 843-848.

Lima LM, Silva Vde A, Palmieri Lde C, Oliveira MC, Foguel D, Polikarpov I. 2010. Identification of a novel ligand binding motif in the transthyretin channel. Bioorganic & medicinal chemistry 18(1): 100-110.
